# BPRS domains, items and subgroups analyses, and CGI-I ratings in pooled data from non-interventional studies of aripiprazole once-monthly in schizophrenia (REACT study)

**DOI:** 10.1186/s12888-023-04651-w

**Published:** 2023-03-14

**Authors:** Daniel Schöttle, Wolfgang Janetzky, Francois Therrien, Klaus Wiedemann

**Affiliations:** 1 Klinik Für Psychiatrie Und Psychotherapie, Zentrum Für Psychosoziale Medizin, Universitätsklinikum Hamburg-Eppendorf, Martinistrasse 52, 20246 Hamburg, Germany; 2 Lundbeck GmbH, Ericusspitze 2, 20457 Hamburg, Germany; 3 Otsuka Canada Pharmaceutical Inc., 2250 Alfred-Nobel Boulevard, Saint-Laurent, Québec, H4S 2C9 Canada

**Keywords:** Schizophrenia, Long-acting injectable, Non-interventional study, Real-world evidence, Aripiprazole once-monthly

## Abstract

**Background:**

Patients with schizophrenia may benefit from treatment with long-acting injectable (LAI) formulations of antipsychotics. Aripiprazole once-monthly (AOM) is an LAI that was tested in two non-interventional studies in Germany and Canada.

**Methods:**

Here, we report on analyses of pooled data from the two non-interventional studies. Patients were treated with AOM under real-life conditions. Data were analyzed for a timeframe of 6 months. We analyzed data on Brief Psychiatric Rating Scale (BPRS) domains and items, BPRS total scores in various patient subgroups (male vs. female patients, patients with disease duration ≤ 5 years and > 5 years, patients with different levels of disease severity at baseline), Clinical Global Impression – Improvement (CGI-I) ratings for the total population and subgroups, and comorbidities for the total population.

**Results:**

Data from 409 patients were included. 65.5% of the patients had comorbidities. Improvements were found in all BPRS domains and items. Furthermore, improvements were similar for male and female patients, patients with disease duration ≤ 5 years and > 5 years, and across different levels of disease severity at baseline. Numerically, more favorable results were found for younger patients, female patients, and those with shorter disease duration.

**Conclusions:**

AOM can be an effective treatment in the broad range of patients, across sexes, regardless of patient age and duration of disease, independently of disease severity, and across symptoms.

**Trial registration:**

NCT02131415 (May 6, 2014), vfa non-interventional studies registry 15960N.

## Background

Antipsychotics are the mainstay of treatment for schizophrenia [1]. However, adherence problems are frequently seen in patients with schizophrenia [2]. Adherence can be improved by use of long-acting injectables (LAI). They offer a reliable means of medication delivery, and, since injections take place at regular intervals during a visit, it is a way to monitor for non-adherence, with a prompt for the treating physician to reach out to any patient who missed their previous scheduled injection [3]. Because of growing evidence for superior real-world effectiveness of LAIs [4, 5], there is a trend to use them early in the course of the disease, rather than as a last resort treatment option. This seems feasible, as a great majority of patients in the early stages of the disease accepted treatment with the LAI aripiprazole once-monthly (AOM) in the PRELAPSE trial, which had a quasi-naturalistic design [6].

AOM is an LAI based on the orally-administered partial D2 agonist aripiprazole. In pivotal randomized controlled trials (RCTs), AOM has been found to be superior to placebo [7] and non-inferior to oral aripiprazole [8]. Additionally, AOM’s effectiveness has been shown in pre-post studies [9, 10], the quasi-naturalistic randomized trial PRELAPSE [11], as well as prospective non-interventional studies in Canada [12] and Germany [13, 14]. In these two non-interventional studies, improvements in symptoms of schizophrenia [12, 13] as well as functional outcomes [12, 14] had been found. In order to validate previous results on AOM effectiveness and safety in a larger population and to improve statistical power for preplanned subgroup analyses, data from both studies were pooled and re-analysed. Results on psychopathology (Brief Psychiatric Rating Scale, BPRS) and disease severity (Clinical Global Impression—Severity, CGI-S) have already been published [15]. Briefly, BPRS global score was 48.1 (SD 15.6) at baseline and reached 36.5 (SD 13.7) at month 6, while CGI-S decreased from 4.47 (SD 0.90) at baseline to 3.64 (SD 1.16) at month 6 [15]. Here, we report additional post-hoc analyses on BPRS domain and items scores, as well as subgroup analyses stratified by sex, disease duration and baseline severity. Clinical Global Impression – Improvement (CGI-I) values and comorbidities are also reported here. Reporting of BPRS domains and items will enable physicians to translate our findings to what they see in everyday practice, where improvement of a single symptom or symptom domain may be more tangible than that of a complex global score. Furthermore, the subgroups reported here were chosen for their clinical relevance. Subgroup analyses like the one reported here are scarce in the literature, which is why our report might be of special interest.

## Methods

This is a post-hoc analysis of pooled data that was originally collected in non-interventional studies conducted in Germany (Verband Forschender Arzneimittelhersteller [vfa] non-interventional studies registry 15960N) [13, 14] and Canada (NCT02131415) [12]. In these studies, patients with schizophrenia who started treatment with AOM according to local product labelling were followed for at least 6 months. According to the local product labels, both in Canada and Germany, tolerability of oral aripiprazole had to be established in patients who had never taken aripiprazole before treatment with AOM was initiated. In addition, the German summary of product characteristics recommends that patients be stabilized on oral aripiprazole before initiation of AOM. Injection intervals were to follow local product labelling, including recommendations on how to deal with missed or delayed injections.

Both studies used similar designs, were performed in similar conditions, and used the same rating scales. Before pooling the results, we undertook a feasibility analysis that compared the baseline data and outcomes of both studies to assess if cofounding factors influenced the results. Descriptive statistical analysis was followed by clinical discussion and decision. We found that pooling the data would produce valid results.

Other studies were not available for pooling, since no other study used a similar study design and studied the same outcomes.

Patients with data at baseline and at least one post-baseline assessment at month 3 or month 6 were included in this analysis. Inclusion and exclusion criteria were similar in the German and Canadian studies and have been reported in detail previously [12, 13]. Inclusion in the original studies was decided only after the treating physician had prescribed AOM.

The BPRS is a clinician-rated score of schizophrenia symptoms that is made up of 18 items in 5 domains. Each item is rated between 1 (symptom absent) and 7 (symptom extremely severe), yielding a total score between 18 and 126. Response on the BPRS was defined as a ≥ 20% reduction in BPRS total score. The BPRS can be grouped into 5 domains which are made up of the following items [16]: The domain anxiety/depression is made up of the items somatic concern, anxiety, guilt feelings, and depressive mood.

The domain anergia is made up of the items emotional withdrawal, motor retardation, blunted affect, and disorientation.

The domain thought disturbance is made up of the items conceptual disorganization, grandiosity, hallucinatory behavior, and unusual thought content.

The domain activation is made up of the items tension, mannerisms and posturing, and excitement.

The domain hostility is made up of the items hostility, suspiciousness, and uncooperativeness.

The CGI-S is a tool that rates the clinician’s impression of illness severity using a single number between 1 (not at all ill) and 7 (extremely ill).

The CGI-I is a tool that rates the clinician’s impression of change in illness severity using a single number between 1 (very much better) and 7 (very much worse).

Data for these endpoints were collected at baseline, month 3 and month 6 in both of the original studies and were used in the present analysis.

A formal rater training was not undertaken, but participating physicians received written instructions on how to score the employed rating scales.

Statistics were mostly descriptive, using means, standard deviations, and proportions. For missing values, imputation was done using the Last Observation Carried Forward (LOCF) method if there was a value for baseline and one for post-baseline (i.e. month 3 or month 6). We decided to employ LOCF because we considered it to be a more conservative estimate of the true clinical development of a patient. No covariates were used. While a mixed model for repeated measures assumes the patient with incomplete data to develop in the same way as the rest of the group under study, LOCF carries the (usually worse) last measured value until the end of the study. Statistical tests were two-sided with alpha = 0.05, without corrections for multiple testing for secondary outcomes.

## Results

### Patient characteristics

Data from 409 patients were analyzed. Patient characteristics are given in Table 1. Most of the patients in the German study (87.9%) had been considered clinically stable by their treating psychiatrists, and they had received pre-treatment with oral aripiprazole for a mean of 9.7 months (Standard deviation [SD], 22.3). In the Canadian study, patients were treated with AOM as per local label. As reported previously, the mean duration of observation was 5.49 months (SD: 1.43) [15]. Three hundred and eighty-four patients (93.9%) completed the studies until month 6 [15]. Reasons for discontinuation during the first 6 months or at month 6 included lack of effectiveness (2.7%), adverse drug reaction (1.7%) or other reasons (7.8%) [15].

**Table 1:** Baseline demographics and clinical characteristics

	Total sample (*n* = 409)
Age (years), mean (SD)	38.9 (14.8)
Sex male, n (%)	245 (59.9)
Body Mass Index (kg/m^2^), mean (SD)	29.2 (6.9)
Age at diagnosis (years), mean (SD)	29.2 (11.7)
Duration of disease since diagnosis (years), mean (SD)	9.8 (10.3)
BPRS total score at baseline, mean (SD)	48.1 (15.6)
CGI-S at baseline, mean (SD)	4.47 (0.90)

*SD* Standard deviation

Before starting AOM treatment, patients had been stabilized with oral antipsychotics as per local label, i.e. oral aripiprazole in Germany or any oral antipsychotic in Canada. The concomitant treatment at study start was oral aripiprazole in 77.3% of the patients. Other oral antipsychotics that more than 2% of the patients used concomitantly were quetiapine (6.4%), olanzapine (5.4%), clozapine (2.9%), and risperidone (2.9%) [15].

The mean AOM dose at study start was 374 mg (SD: 50.5); 315 patients (77.0%) received a dose of 400 mg. Mean doses remained stable throughout the study, with a mean month 6 dose of 375 mg (SD: 73.7). Out of the 316 patients taking concomitant oral aripiprazole, 184 discontinued oral aripiprazole earlier or later than recommended in the product label [15].

### Comorbidities

Many of the patients (65.5%) in our sample had comorbidities at baseline. The most frequent comorbidities are shown in Table 2. Depression was the most frequent comorbidity, affecting 14.9% of the patients.

**Table 2:** Comorbidities at baseline

	Total sample (*n* = 409)
Any comorbidity	65.5%
Depression	14.9%
Hypertension	9.3%
Obesity	7.3%
Anxiety	7.1%
Somnolence	6.6%
Extrapyramidal disorder	5.4%
Blood prolactin increased	5.1%

Comorbidities that were present in at least 5% of the patients are shown. Some patients had multiple comorbidities, with one patient presenting with a total of 13 comorbidities

### BPRS domains

**Fig. 1:**
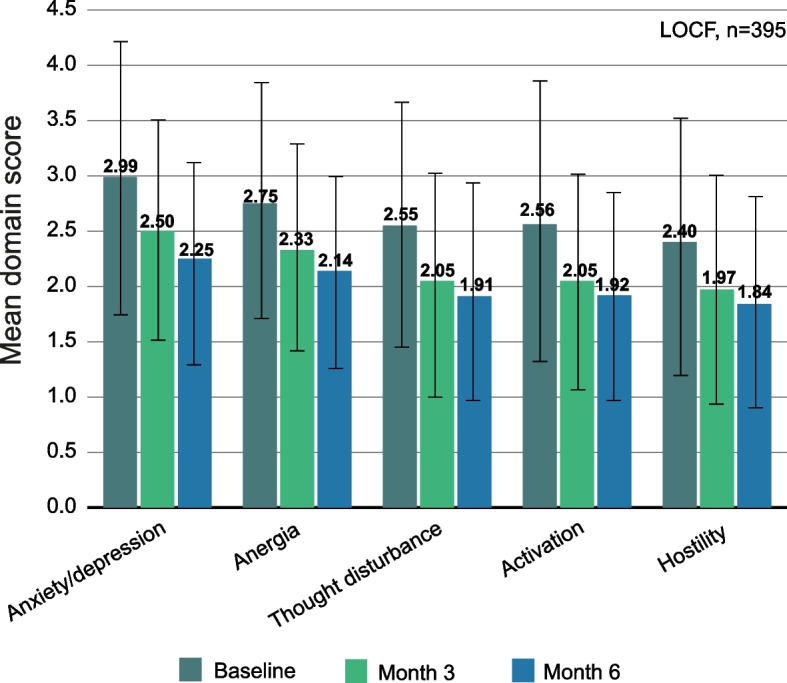
Mean scores of BPRS domains. Error bars represent standard deviations. All month 3 and month 6 reductions are significant compared to baseline (*p* < 0.001). *n* = 395, LOCF

The mean BPRS total score for the total sample was 48.1 (SD 15.6) at baseline and reached 36.5 (SD 13.7) at month 6 [15]. BPRS domain scores are shown in Fig. 1. All domain scores improved significantly during the study (*p* < 0.001). The highest domain scores were found in “anxiety/depression” and the lowest scores in “hostility” throughout the 6 months analyzed. “Anxiety/depression” showed the greatest improvement, with a mean difference from baseline to month 6 of -0.74 (SD, 1.10).

### BPRS items

**Fig. 2:**
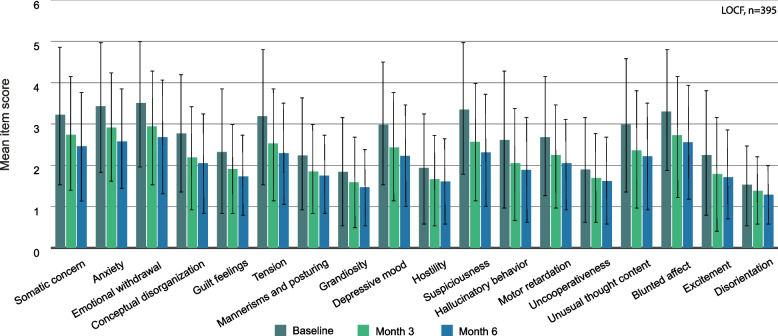
Scores of individual BPRS items. Error bars represent standard deviations. All month 3 and month 6 reductions are significant compared to baseline (*p* < 0.001). *n* = 395, LOCF

BPRS item scores are shown in Fig. 2. All item scores improved significantly during the study. The item “emotional withdrawal” stood out as the one with the highest scores both at baseline (3.51, SD 1.50) and at month 6 (2.68, SD 1.38) of the study. The lowest scores were found in the item “disorientation” (baseline: 1.53, SD 1.02; month 6: 1.29, SD 0.71). The greatest improvement was achieved in “suspiciousness” with a mean difference from baseline to month 6 of -1.04 (SD, 1.59).

### BPRS – subgroup analyses

**Fig. 3:**
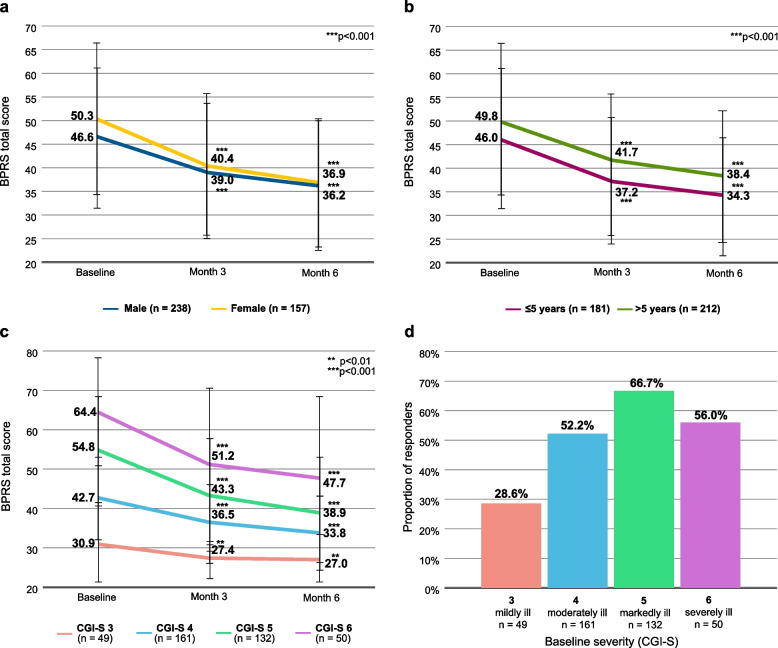
BPRS subgroup analyses. **a** Mean BPRS total score, stratified by sex. **b** Mean BPRS total score, stratified by disease duration. **c** Mean BPRS total score, stratified by disease severity at baseline (represented by CGI-S rating). **d** Treatment response (≥ 20% reduction in BPRS total score) by disease severity at baseline. One patient with CGI-S = 1 and two patients with CGI-S = 7 are not shown in panels **c** and **d**. Error bars represent standard deviations

Subgroup analyses of the BPRS total score are shown in Fig. 3. On average, female patients started out with higher ratings on the BPRS, while both sexes showed almost the same ratings at month 6 (Fig. 3a). Response rates (≥ 20% reduction in BPRS total score) were 50.0% in male patients and 61.2% in female patients.

Patients with a disease duration of more than 5 years had consistently higher BPRS total scores than patients with a disease duration of ≤ 5 years (Fig. 3b). The response rate among patients with a disease duration ≤ 5 years was 54.1%, and for patients with a disease duration of more than 5 years it was 54.3%.

Patients with any baseline disease severity (expressed by baseline Clinical Global Impression – Severity, CGI-S) experienced improvements during the study (Fig. 3c), with the exception of a single patient with baseline CGI-S = 1 (not shown). Statistical significance was not reached for 2 patients with baseline CGI-S = 7 (not shown).

Likewise, responders showing ≥ 20% reduction in BPRS total score were found among patients with any baseline severity, with the highest percentage found in patients with baseline CGI-S = 5 (Fig. 3d).

In additional exploratory analyses, we examined BPRS total score changes in patients with or without adverse events, or with and without medication use errors (which had been coded as adverse events). We found only minimal numerical differences between these groups (data not shown).

### CGI-I

**Fig. 4:**
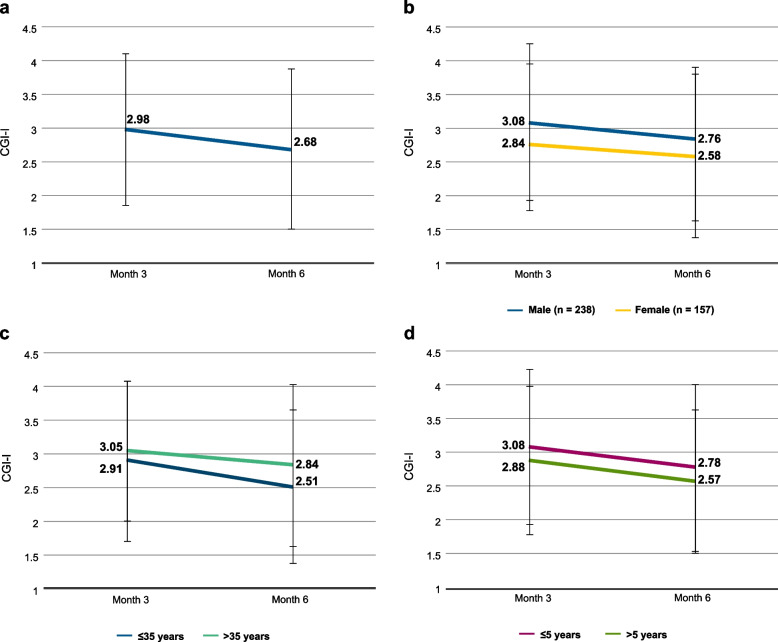
CGI-I. **a** Total sample (*n* = 383 at month 3, *n* = 367 at month 6). **b** CGI-I by sex; male: month 3, *n* = 231; month 6, *n* = 216; female: month 3, *n* = 152; month 6, *n* = 151. **c** CGI-I by age; ≤ 35 years: month 3, *n* = 188; month 6, *n* = 172; > 35 years: month 3, *n* = 195; month 6, *n* = 195. **d** CGI-I by illness duration; ≤ 5 years: month 3, *n* = 178; month 6, *n* = 163; > 5 years: month 3, *n* = 203; month 6, *n* = 203

CGI-I values are shown in Fig. 4. In the total sample, mean CGI-I values corresponded to “minimally improved” at month 3 and “minimally to much improved” at month 6 (Fig. 4a). Female patients tended to do slightly better than male patients (Fig. 4b), younger patients better than older patients (Fig. 4c) and patients with shorter disease duration better than patients with longer disease duration (Fig. 4d).

### Safety

Safety data have been reported previously [15]. Briefly, 192 patients (46.9%) experienced adverse events during the first 6 months of treatment, and 7 patients (1.7%) discontinued because of adverse events. The most frequent adverse events were psychotic symptoms (affecting 7.6% of patients), extrapyramidal symptoms (2.9%), akathisia (2.0%), injection site pain (1.5%) and increased weight (1.5%).

## Discussion

Our analyses show that patients treated with AOM in a real-world setting showed improved symptoms across all domains and in all symptoms studied. Furthermore, similar improvements were seen across sexes, in patients with shorter or longer disease duration, and across different levels of disease severity at baseline. These findings were also reflected in the CGI-I values. We found numerically more favorable results in younger patients, female patients, and those with shorter disease duration. Taken together, it seems that AOM can be effective in the broad range of patients, across sexes, regardless of patient age and duration of disease, independently of disease severity, and across symptoms.

Although many of the patients were pre-treated with oral antipsychotics, and the majority of the German patients were considered clinically stable by their treating clinicians, further improvements were found during 6 months of treatment with AOM. These improvements were observed in all investigated subgroups of patients. This is remarkable, since the expectation would be for the patients to merely remain stable. However, the majority of patients further improved after starting treatment with AOM.

Regarding response, we found most responders in the group of patients that had a baseline severity of CGI-S = 5, and a tendency for more responders in subgroups with higher baseline CGI-S values. This is most likely a floor effect, since patients with less severe disease at baseline had less room for improvement.

We chose a 20% improvement criterion to define a response because many of the patients were pre-treated with oral antipsychotics and the majority of them would not be considered to be acutely ill; in the German subpopulation, the treating physicians were asked whether they considered their patients to be clinically stable, which was the case for 87.9% of the patients [13]. Therefore, we considered a 20% improvement an adequate response here.

Many of the patients under study presented with comorbidities: 65.5% of the patients had at least one documented comorbidity. Many of these patients, especially those with psychiatric comorbidities (i.e. depression and anxiety), would have been excluded from randomized controlled trials on antipsychotic efficacy in schizophrenia, underscoring how important it is to additionally conduct non-interventional studies in order to produce more generalizable results. 14.9% of the patients presented with depression, which is much higher than the 6% that are estimated for the general population [17]; however, depression rates of about 40% have been reported for patients with schizophrenia [18]. The fact that negative symptoms of schizophrenia and depression are very similar and may, in fact, overlap, may make it difficult to make a diagnostic confirmation on presence or absence of depression. Some of the reported comorbidities (somnolence, extrapyramidal disorder, prolactin increase) are typical side effects of antipsychotic drugs, and may in fact have eventually arisen from the previous medication of the patients. However, the decision to report these as comorbidities at baseline was made by each individual treating physician.

Our study has limitations as it is a post-hoc analysis. In the original studies, there was no randomization of patients and there were no control groups. Therefore, confounders cannot be excluded or identified, and a causal relationship of the treatment to our observations cannot be ensured. Participating physicians did not receive a formal rater training, but were given written instructions on how to use the rating scales. Patients may have had expectation bias, since they were aware of the treatment and had voluntarily consented to treatment with AOM. Since many of the patients had been treated with oral aripiprazole before study entry, the sample may be enriched with patients who tolerate aripiprazole. Nevertheless, real-life studies are an important complement to RCTs, because they also include patients with comorbidities and patients that use multiple medications.

## Conclusions

In patients with schizophrenia who were treated with AOM under real-life conditions, we found improvements across symptoms, in both sexes, in older as well as younger patients, in patients with varying duration of disease, and varying degrees of severity at baseline. These findings suggest that patients with schizophrenia who have various different characteristics can still benefit from treatment with AOM.

## Data Availability

The datasets used and analyzed during the current study are available from the corresponding author on reasonable request.

## Abbreviations

AOM: Aripiprazole once-monthly
BPRS: Brief psychiatric rating scale
CGI-I: Clinical Global Impression – Improvement
CGI-S: Clinical Global Impression – Severity
LAI: Long-acting injectable
LOCF: Last observation carried forward
PRELAPSE: Prevention of relapse
RCT: Randomized controlled trial
REACT: Real-world effectiveness of aripiprazole once-monthly
SD: Standard deviation
